# Heat Stress During Gametogenesis Irreversibly Damages Female Reproductive Organ in Rice

**DOI:** 10.1186/s12284-022-00578-0

**Published:** 2022-06-28

**Authors:** Wanju Shi, Juan Yang, Ritesh Kumar, Xinzheng Zhang, Somayanda M. Impa, Gui Xiao, S. V. Krishna Jagadish

**Affiliations:** 1 College of Agronomy, Hunan Agricultural University, Changsha, 410128 Hunan China; 2 State Key Laboratory of Hybrid Rice, Hunan Hybrid Rice Research Center, Changsha, 410125 China; 3 Department of Agronomy, Kansas State University, Manhattan, KS 66506 USA; 4 International Rice Research Institute, DAPO Box 7777, Metro Manila, Philippines; 5 Department of Plant and Soil Science, Texas Tech University, Lubbock, TX 79409-2122 USA

**Keywords:** Heat stress, Megasporogenesis, Metabolites, Ovary, Pistil, Rice, Spikelet fertility

## Abstract

Heat stress during gametogenesis leads to spikelet sterility. To ascertain the role of female reproductive organ (pistil), two rice genotypes N22 and IR64 with contrasting heat stress responses were exposed to control (30 °C) and heat stress (38 °C and 40 °C) during megasporogenesis. Anatomical observations of ovule revealed greater disappearance of megaspore mother cell and nuclei at early stages, and during later stages mature embryo sac without female germ unit, improper positioning of nuclei, and shrunken embryo sac was observed in the sensitive IR64. Under heat stress, a decrease in sugar and starch, increase in H_2_O_2_ and malondialdehyde with lower antioxidant enzyme activities were recorded in pistils of both N22 and IR64. Lower accumulation of TCA cycle metabolites and amino acids were noticed in IR64 pistils under heat stress at gametogenesis, whereas N22 exhibited favorable metabolite profiles. At heading, however, N22 pistils had higher carbohydrate accumulation and better ROS homeostasis, suggesting higher recovery after heat stress exposure. In summary, the results indicate that heat stress during megasporogenesis leads to irreversible anatomical and physiological changes in pistil and alters metabolic signatures leading to increased spikelet sterility in rice. Mechanisms identified for enhanced heat tolerance in pistil can help in developing rice varieties that are better adapted to future hotter climate.

**Supplementary Information:**

The online version contains supplementary material available at 10.1186/s12284-022-00578-0.

## Background

Higher ambient temperatures are becoming a primary factor limiting crop growth and productivity (Vogel et al. 2019). Rice (*Oryza sativa* L.), a major source of calories for more than half the world’s population, is shown to be highly sensitive to heat stress in major rice growing regions in China (Lv et al. 2018), Laos and southern India (Ishimaru et al. 2016), Japan (Hasegawa et al. 2009) and Africa (van Oort and Zwart 2018), posing a serious threat to sustain global rice production. In addition, climate modelling studies project a steady rise in mean temperature throughout the twenty-first century, accompanied with more frequent and intense heat stress events both at the regional and global scales (IPCC 2014; Basha et al. 2017). To respond to rapidly changing temperature scenarios, identifying traits and exploring mechanisms that enhance heat tolerance at critical developmental stages in rice is essential and timely.

Heat stress sensitivity of rice varies depending on the developmental stage. Gametogenesis and flowering have been identified to be the two most sensitive stages to heat stress (Jagadish et al. 2014; Prasad et al. 2017). In response to the anticipated heat stress scenarios, efforts have been intensified to quantify heat stress impact during flowering in rice (Shi et al. 2018; Jagadish 2020), while the gametogenesis stage has received limited attention. Heat stress coinciding with microspore stage of pollen development is shown to result in structural and functional abnormalities in the male reproductive organ, subsequently reducing pollen viability and increasing spikelet sterility (Sage et al. 2015; Lohani et al. 2020). The reduction in pollen viability under heat stress has been attributed to disruption of meiotic division during pollen development, degeneration of tapetal cells (Endo et al. 2009), disturbed carbohydrate metabolism and oxidative damage (Li et al. 2015; Zinta et al. 2016). However, the influence of heat stress during gametogenesis on female reproductive organ development and viability in rice is not well understood.

Spikelet fertility is determined by a combination of pistil and pollen viability. Comparatively, the male gametophyte is considered to be more sensitive than the female gametophyte (Jagadish 2020)*,* indicating that pollen viability plays a more important role in determining spikelet fertility on exposure to heat stress. However, abnormal embryo sac was the primary reason for reduced seed set in wheat (*Triticum aestivum* L.), although unstressed pollens were used to pollinate heat stressed pistils (Saini et al. 1983). Similarly, reduced seed-set as a result of loss in female organ viability under heat stress exposure has been observed in canola (*Brassica napus* L, Polowick and Sawhney 1988), sorghum ([*Sorghum bicolor* (L.) Moench], Djanaguiraman et al. 2018a) and pearl millet (*Pennisetum glaucum* L. R. Br., Djanaguiraman et al. 2018b). In rice, a cross pollination exercise demonstrated that the female reproductive organ was unaffected even at 41 °C during flowering, while the loss in pollen viability induced significant spikelet sterility from 35 °C (Satake and Yoshida 1978). Similarly, Endo et al. (2009) pollinated rice pistils exposed to heat-stress during early microspore stage with non-stressed pollen and concluded that the stigma receptivity to pollen was unaffected. However, this study considered just the stigma receptivity but did not account for additional processes such as variations in embryo sac that determine pistil’s vulnerability to heat stress.

In a recent review, Wang et al. (2021), have discussed the varying degrees of sensitivity of pistils to heat stress depending on the species and developmental stage. Exposure of wheat to 30 °C for three days at the onset of meiosis resulted in reduced embryo sac size and negatively affected nucellus development (Saini et al. 1983). Tomato (*Solanum tuberosum* L.) plants exposed to 40 °C for 3 h on two consecutive days starting nine or six days prior to flower opening resulted in ovules with degenerated megaspores or degenerated egg cell and polar nuclei (Iwahori 1966). In sorghum, heat stress reduced the size of the transmitting style tissue, leading to desiccated and flaccid stigma, style, and ovary (Djanaguiraman et al. 2018a). Further, heat sensitive sorghum genotype had damaged ovarian tissue near micropylar region, condensed cytoplasmic contents and disintegrated nucleolus and nucleus, but these impacts were significantly lower in the tolerant genotype (Chiluwal et al. 2020). In sweet cherry (*Prunus avium* L.) and pea (*Pisum sativum*), ovaries with higher callose deposition were observed after exposure to heat stress (Zhang et al. 2018; Jiang et al. 2019). Although several studies have observed morphological and anatomical changes in pistils under heat exposure (Lohani et al. 2020; Wang et al. 2021), information on heat stress impacts on ovule development and associated mechanisms are limited in crops and unexplored in rice.

In plants, optimal ROS (Reactive Oxygen Species) levels and antioxidant enzyme activities are required for normal development of reproductive organs and pollen-pistil interactions during flowering (Sharma and Bhatla 2013; Zinta et al. 2016). In rice, heat stress during meiosis resulted in higher ROS accumulation and lower activity of antioxidant enzymes in developing anther, inducing sterility (Zhao et al. 2018). Similarly, heat-stressed pistils of cotton (*Gossypium hirsutum*) and sorghum recorded increased ROS biosynthesis and reduced ROS scavenging capacity, increasing female gametophyte sensitivity to stress-induced ROS accumulation (Snider et al. 2011; Djanaguiraman et al. 2018a). In cotton pistils, heat stress reduced total soluble carbohydrate and adenosine triphosphate (ATP) concentrations (Snider et al. 2011). In addition, multiple levels of metabolic and transcriptomic changes were noticed in rice anther and pistil in response to combined heat and drought stress at flowering (Li et al. 2015). However, similar metabolic changes, ROS and sugar levels in rice pistils exposed to heat stress during pistil development (megasporogenesis) and their response after stress release is not known.

Hence, in our study, we used two rice genotypes contrasting for heat tolerance and exposed them to control temperature and two levels of daytime heat stress during megasporogenesis. Anatomical, biochemical and metabolic analyses were conducted to (1) systematically quantify the contribution of female reproductive organ towards heat stress induced spikelet sterility and (2) ascertain whether the impact of heat stress during gametogenesis on female reproductive organ viability was reversible or irreversible.

## Results

### Spikelet Fertility

**Fig. 1:**
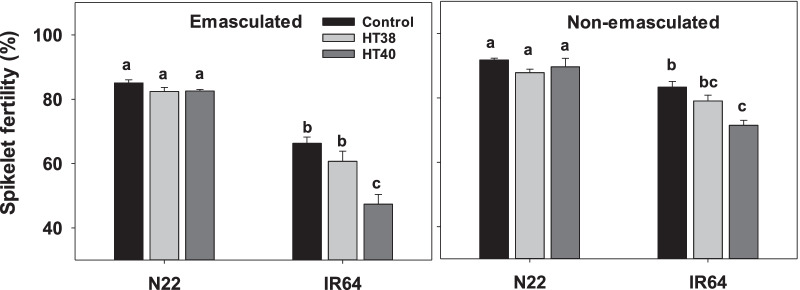
Effects of control (30 °C) and heat stress (38 °C—HT38; 40 °C—HT40) exposure during gametogenesis on spikelet fertility in N22 and IR64. Emasculated – refers to spikelet fertility in panicles in which anthers were manually removed and pollinated by dusting fresh pollen. Non-emasculated - refers to spikelet fertility in panicles with natural pollination i.e., without manual interference. Values shown are mean ± standard error. Means followed by a common letter are not significantly different between temperature treatments and genotypes within either emasculated /non-emasculated plants at *P* ≤ 0.05

Spikelet fertility was estimated from the tagged panicles which were exposed to heat stress (38 °C or 40 °C) or control temperatures during gametogenesis. Panicles which were emasculated and not pollinated manually had zero spikelet fertility in both the genotypes (data not shown), indicating the effectiveness of the emasculation procedure. A reduction in spikelet fertility was observed in plants where pollen grains were manually dusted on control pistils compared to non-emasculated plants, with the reduction being more conspicuous in IR64 (Fig. 1). To account for the observed variation in spikelet fertility, treatment with emasculation followed by manual dusting of fresh pollen on pistils under control conditions was considered as “true” control for making realistic comparison with stress treatments. A significant effect of treatment (T), genotype (G), and T × G interaction effects (*P* < 0.001) were recorded on spikelet fertility. In both emasculated and non-emasculated panicles of N22, 38 and 40 °C did not induce significant decline in spikelet fertility compared to control (Fig. 1). However, both 38 and 40 °C induced a reduction in spikelet fertility in both emasculated and non-emasculated panicles of IR64, with significantly (*P* < 0.001) lower spikelet fertility under 40 °C compared to control. In summary, the results above capture the negative impact of heat stress on female reproductive organ development leading to increased spikelet sterility in sensitive rice genotype.

### Ovule Anatomy

**Fig. 2:**
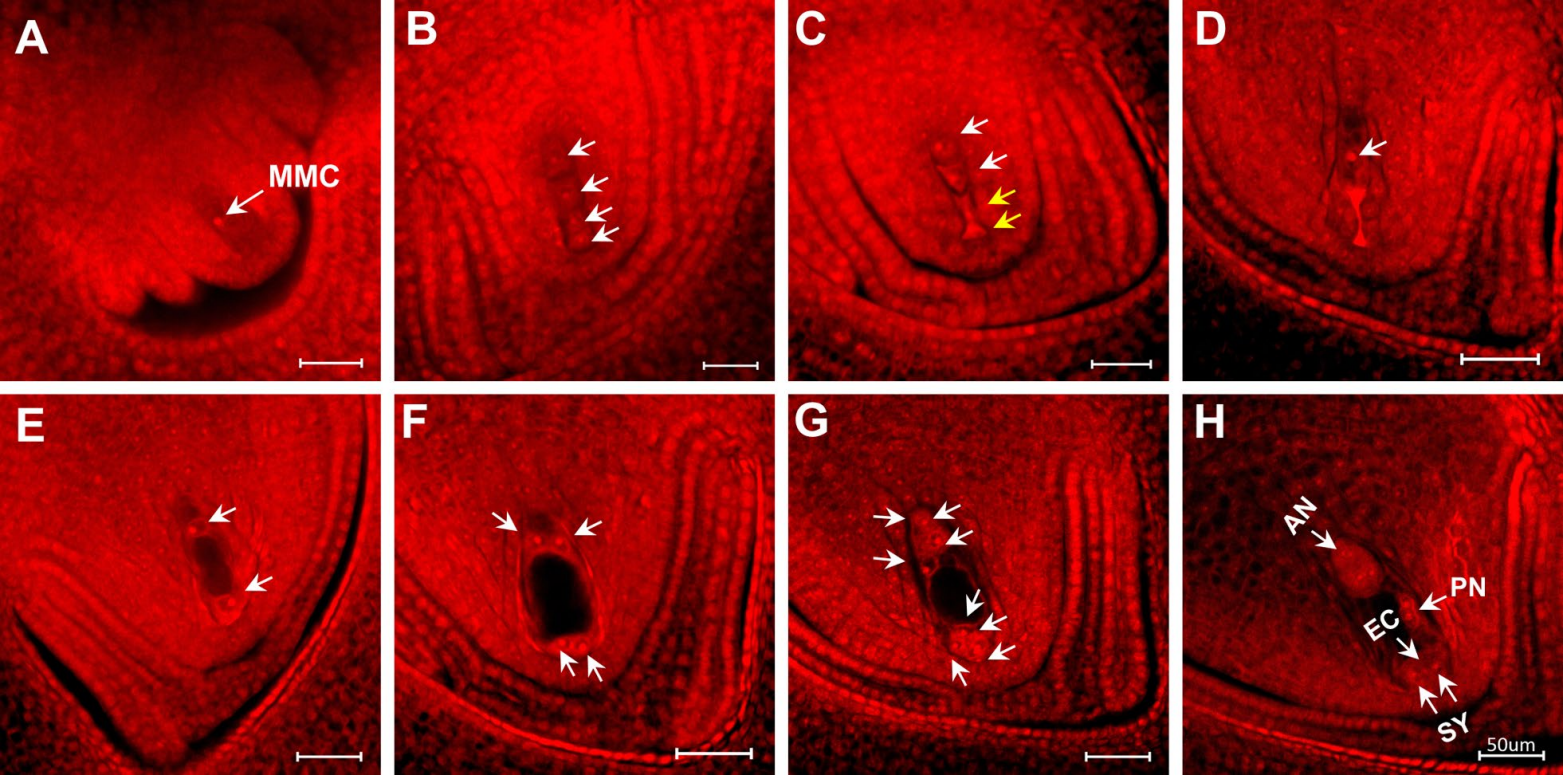
Early embryo sac development in IR64 plants exposed to control temperature (30 °C) during gametogenesis stage for 6 days. AN, antipodal cells; EC, egg cell; MMC, megaspore mother cell; PN, polar nucleus; SY, synergid. (**A**) A rectangle-like megasporocyte with inner and outer integuments; (**B**) Four megaspores linearly arranged after meiosis; (**C**) Degeneration of two megaspores (yellow arrow indicates the degenerated megaspores and white arrow shows active megaspores); (**D**) Degeneration of three megaspores (white arrow indicates the remaining functional megaspore); (**E**) A two-nucleate embryo sac; (**F**) A four-nucleate embryo sac; (**G**) An early stage eight-nucleate embryo sac; and (**H**) Middle stage of eight-nucleate embryo sac development, two polar nuclei have migrated to the central region and egg cell located near the micropylar region

The primary aim was to ascertain the impact of heat stress during the critical tetrad phase, hence the focus of the comparison was between 5 mm (IR64) and 3 mm (N22) based on the findings from Jagadish et al. (2014). Data from other spikelet sizes was collected to determine the proportion of sterility induced by heat stress coinciding with stages before or after tetrad phase. Under control temperature (30 °C) enlarged and differentiated megaspore mother cell (MMC) was accompanied with well differentiated inner and outer integuments in 5 mm and 3 mm spikelets of IR64 (Fig. 2A) and N22 (Additional file 1: Table S1), respectively. In concurrence of elongation of both outer and inner integuments, meiosis in megaspore mother cell resulted in four linearly arranged megaspores in the micropyle-chalaza direction (Fig. 2B). Soon after, three megaspores near the micropyle degenerated and only the chalazal megaspore remained active (Fig. 2C, D). After meiosis, the functional megaspore undergoes mitotic nuclear division to become a two-nucleate cell (Fig. 2E), followed by a second mitotic division to form a four-nucleate cell accompanied with a large vacuole (Fig. 2F). Further, the eight-nuclei sac is formed after the third mitotic division (Fig. 2G), finally resulting in a mature eight-nucleate sac with appropriate positioning of the nuclei (Fig. 2H).

**Fig. 3:**
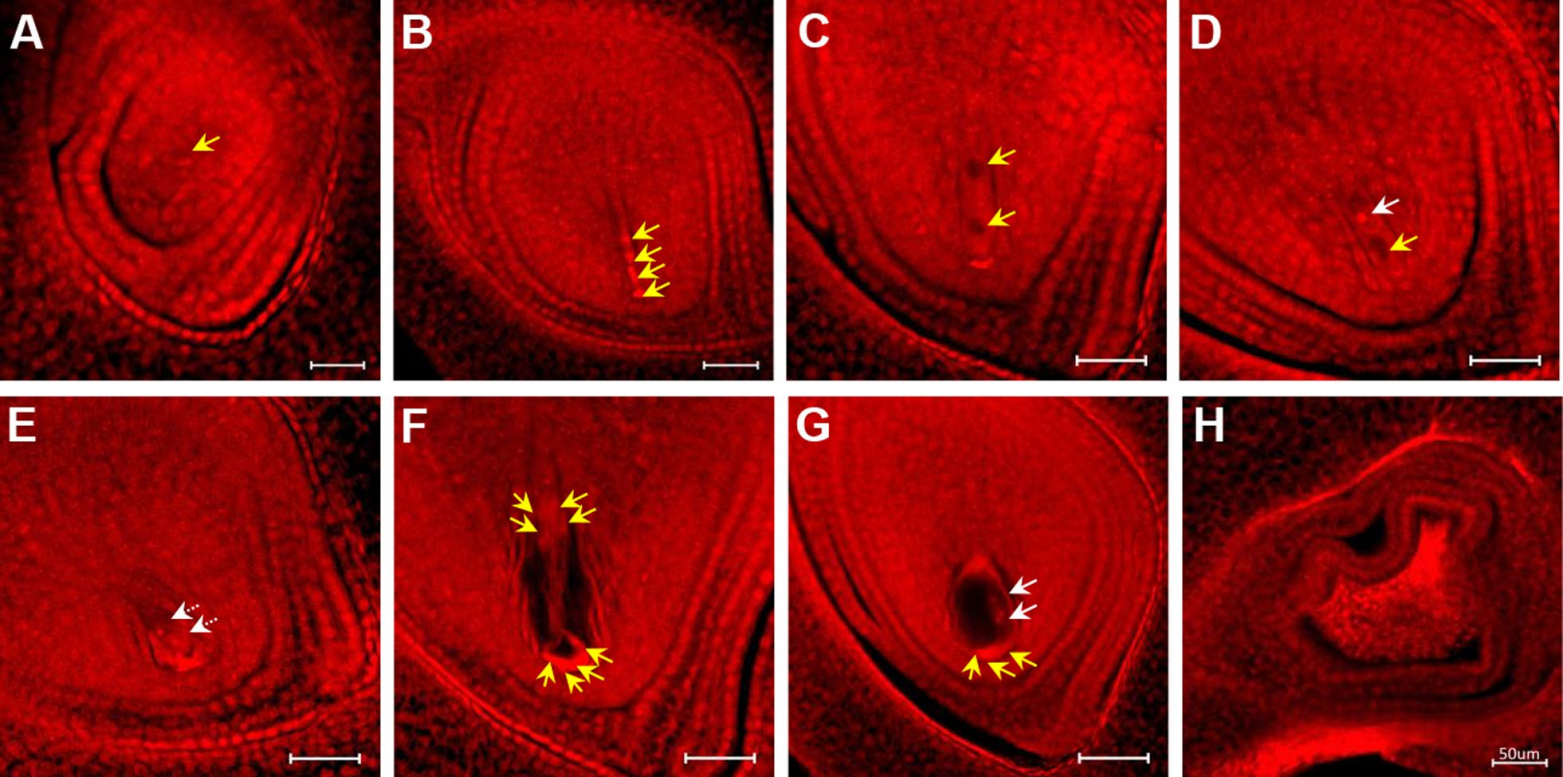
Different types of abnormalities that occurred during embryo sac development in IR64 exposed to six days of heat stresses (38 °C—HT38; 40 °C—HT40) during gametogenesis. (**A**) Degenerated megaspore mother cell (yellow arrow indicates the missing nuclei); (**B**) All four megaspores degenerated, indicated by yellow arrows; (**C**) Abnormal two-nucleate embryo sac, with both nuclei degenerated (yellow arrows); (**D**) Abnormal two-nucleate embryo sac with one nucleus degenerated (yellow arrow), white arrow shows the remaining one active nucleus. (**E**) Abnormal two-nucleate embryo sac without a large vacuole and both nuclei located at the micropylar region (Two nuclei indicated by white arrows were supposed to be located one each at micropylar and chalaza end, similar to Figure 2E). (**F**) Abnormal eight-nucleate embryo sac with missing nuclei (yellow arrows); (**G**) Abnormal eight-nucleate embryo sac with degenerated egg apparatus (yellow arrows indicate the degenerated egg cell and synergids); white arrow indicates two active nuclei; and (**H**) Shrunken embryo sac

On heat stress exposure, a higher frequency of abnormalities were developed in embryo sacs compared to control in both genotypes, with a significantly higher frequency of abnormalities in IR64 (reduced to 82.5% of normal embryo sacs at 40 °C) than N22 at 40 °C (Fig. 3 and Table 1). At the initiation of megasporogenesis, degeneration of MMC (Fig. 3A) was observed in both N22 and IR64 spikelets (Table 1). Immediately following the meiosis of MMC, heat stress resulted in degeneration of all four megaspores instead of just three nuclei as seen under control conditions (Fig. 3B). This phenomenon (degeneration of all four megaspores) was more frequent in IR64 only at 40 °C (Table 1). Following meiosis some spikelets had missing nuclei and improperly positioned nuclei under heat stress (Fig. 3C–G) despite having normal morphological structure. In addition, it was observed that some ovaries in IR64 spikelets were completely shrunken and dead (Fig. 3H) when exposed to 40 °C (1.4% at 40 ^o^C, Table 1).

**Table 1:** Percentage abnormalities in the embryo sac samples of N22 and IR64 at six days after exposure to control (30 °C) and heat stresses (38 °C -HT38; 40 °C -HT40) during gametogenesis and heading stage (at recovery)

Stage of observed samples	Percent abnormalities	N22			IR64		
		Control	HT38	HT40	Control	HT38	HT40
Developing embryo sac (spikelets) at six days after exposure to temperature treatments	Number of observed samples	275	311	298	333	329	348
	Embryo sac. with normal developing structure (%)	92.36	89.39	90.60	92.19	88.45	82.47
	With degenerated megaspore mother cell (%)	0.73	1.93	0.34	1.20	2.74	1.72
	With degenerated all four megaspores (%)	5.45	5.47	6.38	5.41	5.17	10.06
	Abnormal two-nucleate embryo sac (%)	1.09	2.57	1.68	0.90	1.22	2.87
	Abnormal eight-nucleate embryo sac (%)	0.00	0.64	0.34	0.00	2.13	1.44
	Shrunken embryo sac (%)	0.36	0.00	0.67	0.30	0.30	1.44
Mature embryo sac (Spikelets) at heading stage	Number of observed samples	113	116	108	107	183	152
	Mature embryo sac. with normal structure (%)	95.58	93.37	94.44	96.26	85.25	83.55
	Degenerated embryo sac (%)	0.00	0.86	0.93	0.00	0.55	1.32
	With degenerated both egg cell and polar nuclei (%)	1.77	1.72	0.93	1.87	6.01	6.58
	With degenerated egg cell (%)	1.77	1.72	2.78	1.87	7.10	7.24
	With improperly positioned egg cell and/or polar nuclei (%)	0.88	1.72	0.93	0.00	1.09	1.32

Data presented is the summary of heat stress induced damage across all spikelet sizes, with specific spikelet size details provided in Additional file 1: Table S1

**Fig. 4:**
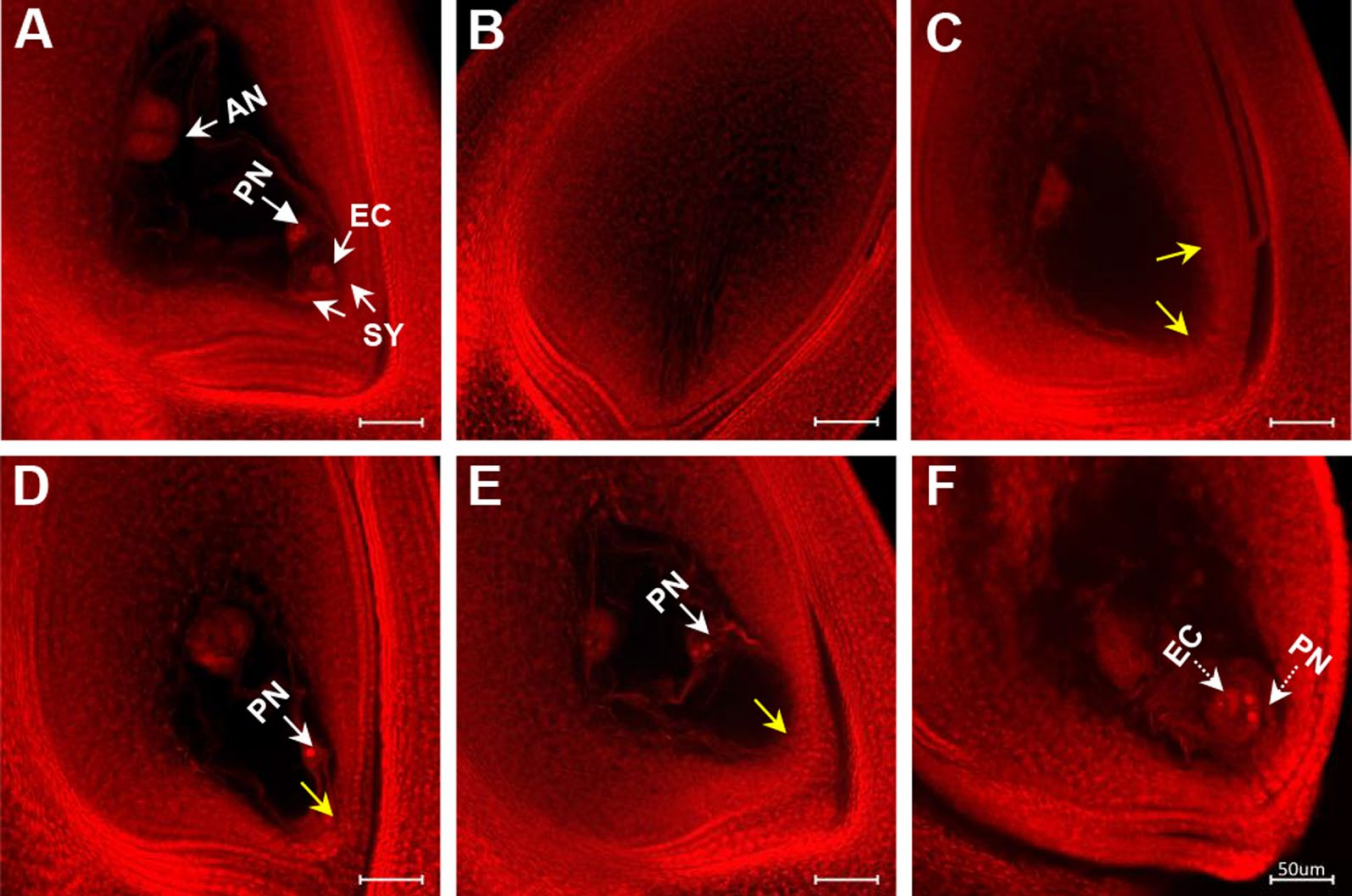
Mature embryo sac at heading stage (post-stress recovery) in IR64 plants exposed to six days of control (30 °C, panel **A**) and heat stress (38 °C—HT38; 40 °C—HT40, panels **B** to **F**) during gametogenesis. (**A**) A normal mature embryo sac with antipodal cells (AN), two polar nuclei (PN), two synergid cells (SY) and one egg cell (EC) under control (30 ℃); (**B**) Degenerated embryo sac, without differentiation of embryo sac cavity; (**C**) Abnormal embryo sac without female germ unit, including egg cell and polar nuclei (indicated by yellow arrows); (**D**) Abnormal embryo sac without egg cell (yellow arrow); (**E**) Abnormal embryo sac without egg cell (yellow arrow) and two polar nuclei located far from female units (white arrow); and (**F**) Egg cell and polar nuclei were at the wrong position (two polar nuclei were closer to micropylar region than the egg cell)

To gain information on the female reproductive organ’s ability to recover after heat stress was released, ovaries at heading were observed. At the heading stage, under control (30 °C) the mature embryo sac consisted of one egg cell located in the receptacle end and two polar nuclei in the middle which subsequently develops into embryo and endosperm, respectively (Fig. 4A). Under heat stress, in some ovaries the entire embryo sac was completely degenerated in IR64 at 38 °C and 40 °C (Fig. 4B). In some cases, the embryo sac was without the egg apparatus, i.e. egg cell and/or polar nuclei (Fig. 4C–E) or with improperly positioned nuclei (Fig. 4E, F).

Pistils observed at heading showed large increases in the frequency of abnormality in mature embryo sac in IR64 (normal embryo sac reduced to 83.6% at 40 °C) while more than 94% of embryo sacs were normal in N22 after heat exposure (Table 1). Completely degenerated embryo sacs increased in both genotypes, particularly in IR64 pistils at 40 °C. The frequency of embryo sac with degenerated egg cell and/or polar nuclei was higher in IR64 (between 6.01 and 7.24%) compared to N22 (between 0.93 and 2.78%) under different levels of heat stress. Moreover, embryo sac with improperly positioned egg cell and/or polar nuclei increased under stress, in both N22 and IR64 (Table 1).

### Total Soluble Sugar and Starch

**Fig. 5:**
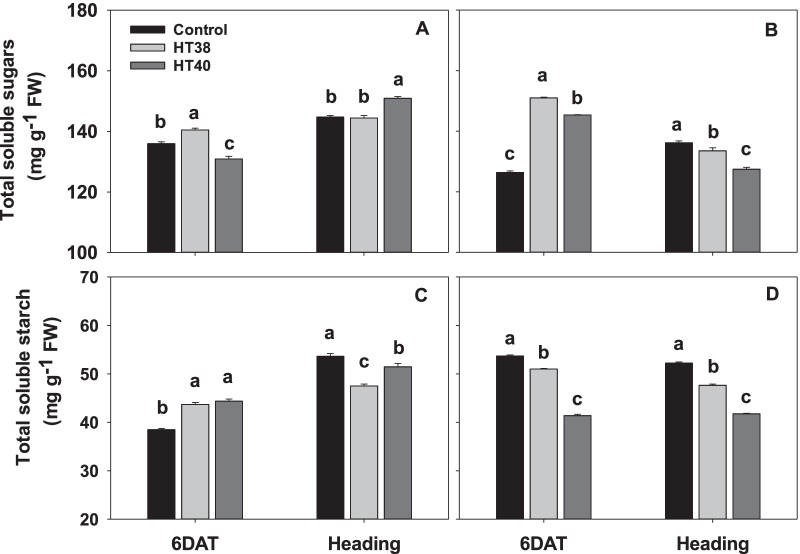
Total soluble sugars and starch in pistils of N22 (**A** and **C**) and IR64 (**B** and **D**) plants at six days of control (30 °C) and heat stress (38 °C—HT38; 40 °C—HT40) exposure during gametogenesis and recovery at heading. Bars with common letter are not significantly different between temperature treatments in a genotype at *P* ≤ 0.05. 6DAT - 6 days after treatment

Significant (*P* < 0.001) T, G, growth stage (S) and their interaction effects were recorded for both total soluble sugars and starch in pistils (Fig. 5; Additional file 2: Table S2). Total soluble sugars increased significantly at 6 days of heat stress, compared to control in both the genotypes except for N22 at 40 °C. However, at heading i.e., after recovery, total soluble sugar increased significantly only at 40 °C in N22, while a significant decline was observed at both levels of stress in IR64 (Fig. 5A, B). Soluble starch concentration increased significantly in N22 at 6 days of heat stress (both 38 °C and 40 °C) compared to control. However, in IR64 soluble starch significantly decreased by 5% at 38 °C and with a much larger decline of 23% at 40 °C compared to control at 6 days after treatment (DAT). At heading, starch concentration was significantly lower in pistils obtained from plants exposed to heat stress (38 and 40 °C) in both the genotypes, with a much higher decline in IR64 at 40 °C (20.3%) (Fig. 5C, D).

### Oxidative Damage and Antioxidant Enzyme Activities

**Fig. 6:**
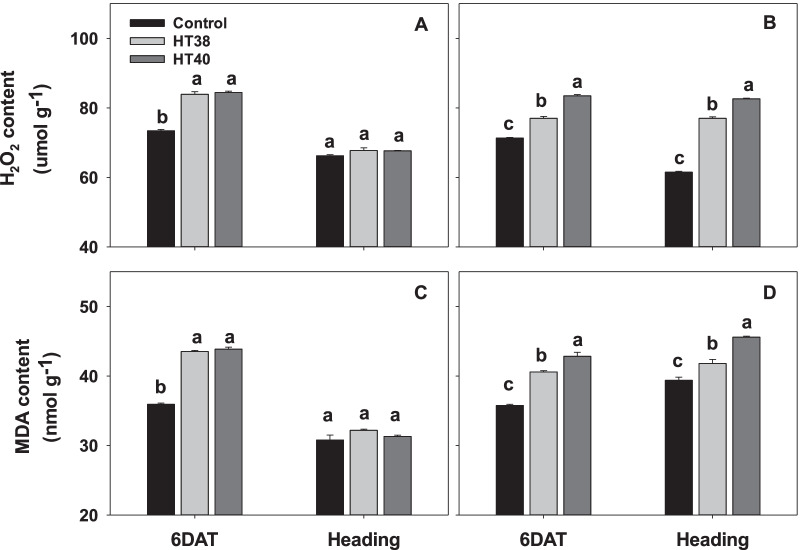
Hydrogen peroxide (H_2_O_2_) and malonaldehyde (MDA) in pistils of N22 (**A** and **C**) and IR64 (**B** and **D**) at six days after treatment (6DAT) under control and heat stress (38 °C -HT38; 40 °C—HT40) at gametogenesis and recovery i.e., at heading. Values shown are mean ± standard error (n=3). Bars with common letter are not significantly different between temperature treatments in a genotype at *P* ≤ 0.05. 6DAT - 6 days after treatment

**Fig. 7:**
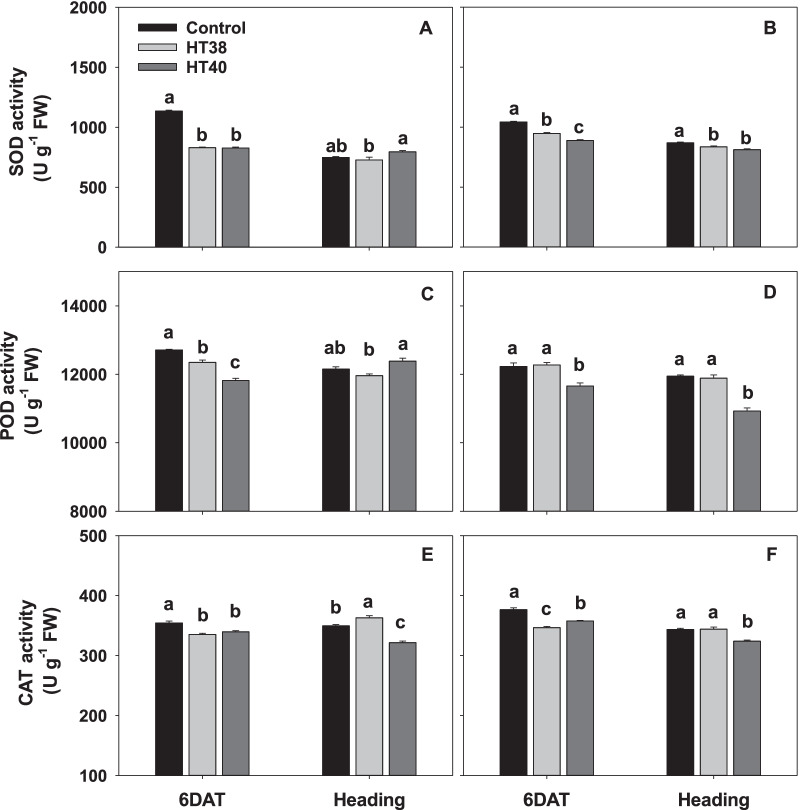
Changes in antioxidant enzymatic activities in pistils of N22 (**A**, **C** and **E**) and IR64 (**B**, **D** and **F**) exposed to six days of control and heat stress (38 °C—HT38; 40 °C—HT40) treatments at gametogenesis and recovery at heading stage. Values shown are mean ± standard error (n=3). Bars with common letter are not significantly different between temperature treatments in a genotype at *P* ≤ 0.05. SOD, Superoxide dismutase; POD, Peroxidase; CAT, Catalase. 6DAT - six days after treatment

The H_2_O_2_ and MDA quantified through estimation of TBARS revealed that the oxidative damage was significantly increased under heat stress (38 and 40 °C) in both the tolerant N22 and sensitive IR64, compared to control at 6DAT (Fig. 6). On average, N22 recorded 14.6 and 21.7% increase in H_2_O_2_ and MDA respectively, from both the heat stress levels, whereas IR64 recorded an average increase of 12.5% in H_2_O_2_ and 16.5% in MDA under both the heat stress levels. However, at heading i.e., after post-stress recovery, a significant increase in H_2_O_2_ (on average 29.8% increase at both heat stress levels, Fig. 6B) and MDA (on average 10.9% increase at both stress levels, Fig. 6D) were observed only in IR64, while the H_2_O_2_ and MDA were similar across control and heat stress treatments in N22 (on average 2.2 and 3.0% increase at both the heat stress levels for H_2_O_2_ and MDA, respectively, Fig. 6A, C). At 6 days of heat stress, superoxide dismutase (SOD), peroxidase (POD) and catalase (CAT) activities were significantly declined in both the genotypes (Fig. 7) except for POD activity in IR64 at 38 °C (Fig. 7D). However, in N22 a non-significant change in SOD and POD activities was observed in pistils after recovery (at heading) compared to control (Fig. 7A, C), while CAT activity significantly increased at 38 °C (Fig. 7E). In IR64, SOD activity continued to be significantly lower after recovery (Fig. 7B), while POD and CAT activities reduced only at 40 °C compared to control (Fig. 7D, F).

### Metabolic Profiling

**Fig. 8:**
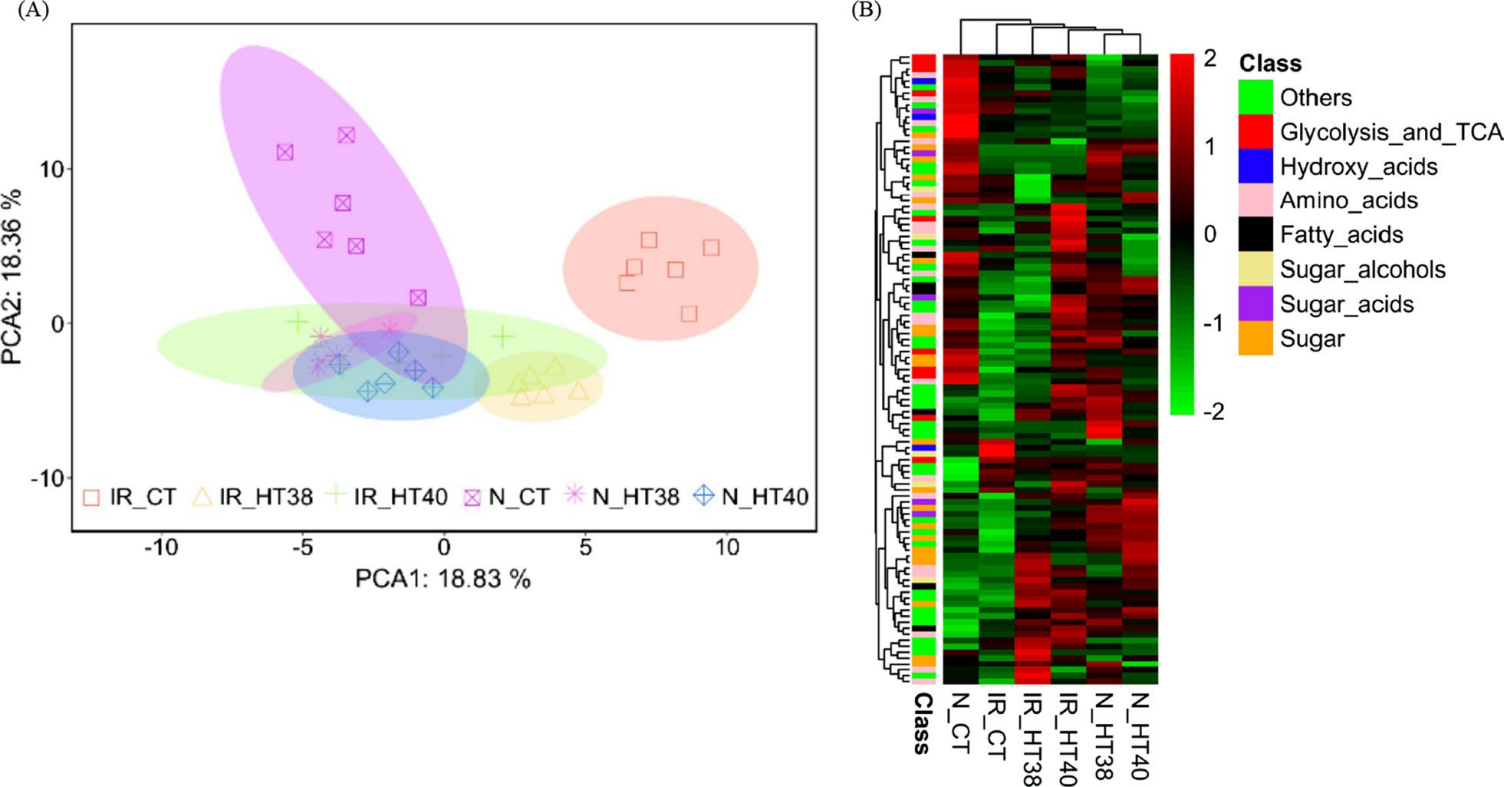
**A** Principal components analysis (PCA) score plots of metabolite data to depict group divergence between cultivars and temperature treatments. **B** Hierarchical clustering through heat map illustrating the levels of metabolites under control and heat stress treatments (distance measure—Euclidean; clustering algorithm-Ward). Values are means of six biological replicates. N, N22; IR, IR64; CT, exposure to temperature at 30 ℃; HT38, exposure to heat stress at 38 °C; HT40, exposure to heat stress at 40 °C. Green color with scale –2 represents lower accumulation of metabolite, whereas red color with scale +2 indicates higher accumulation of metabolite levels in each genotype and temperature treatment combination

Metabolite profiling of pistils at the end of temperature treatment (6DAT) in N22 and IR64, detected a total of 105 pistil metabolites (Additional file 3: Table S3). PCA was performed with the complete metabolite dataset to obtain an overview of pistil metabolites distribution across control (30 °C) and heat stress (38 °C and 40 °C) treatments (Fig. 8A). In total, PC1 and PC2 together contributed more than 37.2% of the total variance. Score plots revealed that in N22, control and heat stress samples grouped together, whereas in IR64, metabolites at 38 °C and 40 °C exposed pistils diverged significantly from control plants (Fig. 8A). The hierarchical clustering of genotypes and treatments based on all the detected metabolites are presented in Fig. 8B.

A two-way ANOVA, with significant genotype (G), temperature (T) and their interaction effects on 105 metabolites is presented in Additional file 4: Table S4. Irrespective of the genotype, a total of 59 metabolites varied with a significant temperature treatment (*P* ≤ 0.05). These 59 metabolites included twelve sugars, nine amino acids, six Glycolysis and TCA related compounds, four sugar acids and twenty-eight others, including hydroxy acids and their derivatives, organic acids and their derivatives, alcohols and polyols. Significant G × T interaction effects (*P* ≤ 0.05) was recorded with 52 metabolites including ten amino acids, eleven sugars and seven Glycolysis and TCA cycle related compounds, two sugar acids, twenty-two others (Additional file 4: Table S4).

**Fig. 9:**
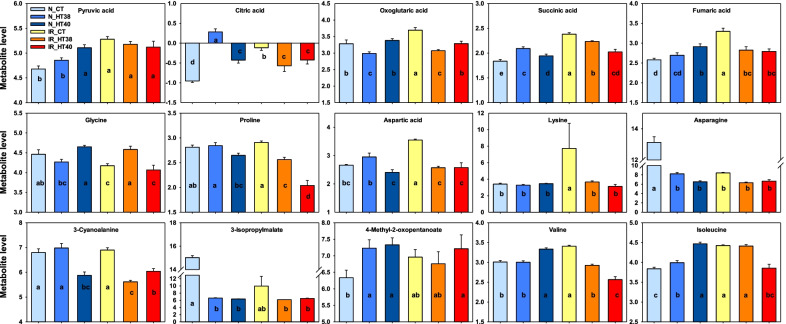
The differential accumulation of pistil metabolites detected in the five highly altered metabolic pathways (including TCA cycle and amino acid pathways) among the two rice genotypes (N22-N, IR64-IR) exposed to control (control-CT) and two heat stress treatments (38 °C—HT38 and 40 °C—HT40) at six days after temperature treatment (6DAT). Bars represent Log2 fold change of the normalized values averaged across replications, bars indicate SE (n = 6). Different alphabets indicate significant difference between temperature treatments and genotypes for a metabolite at 5% LSD

Significantly (*P* ≤ 0.05) altered metabolisms based on pathway enrichment analysis and their impact values are presented in Additional file 5: Table S5. A total of 14 metabolic pathways were significantly (*P* ≤ 0.05) altered across two genotypes and three temperature treatments. The highly altered metabolic pathways were (1) Alanine, aspartate and glutamate metabolism; (2) Citrate cycle (TCA cycle); (3) Valine, leucine and isoleucine biosynthesis; (4) Aminoacyl-tRNA biosynthesis; and (5) Cyanoamino acid metabolism (Additional file5: Table S5). In addition, the metabolites enriched in the above five metabolic pathways are presented in Fig. 9. Under control IR64 accumulated significantly higher concentration for most of the metabolites than N22, except for glycine and asparagine which were significantly lower in IR64 than N22 (Fig. 9). At 6 DAT in N22, all the metabolites from TCA cycle (pyruvic acid, critic acid, oxoglutaric acid, succinic acid, fumaric acid) increased under 38 °C and 40 °C compared to control except for oxoglutaric acid at 38 °C (Fig. 9). However, in IR64 except for the non-significant change in pyruvic acid, other four TCA cycle metabolites recorded a significant reduction under heat stress compared to control. For the metabolites from other four metabolic pathways, 3-Isopropylmalate and asparagine significantly decreased, and 4-Methyl-2-oxopentanoate and isoleucine increased significantly under heat stress compared to control in N22. Remaining metabolites had non-significant changes after exposure to heat stress except for 3-cyanoalanie and valine at 40 °C (Fig. 9). However, in IR64, except for the significant increase in glycine at 38 °C and significant decrease in isoleucine at 40 °C, 5 metabolites (3-Cyanoalanine, proline, aspartic acid, lysine and valine) significantly reduced and remaining 3 metabolites had non-significant changes under heat stress compared to control (Fig. 9).

## Discussion

### Heat Stress Damages Female Reproductive Organ in Rice

Heat stress sensitivity of female reproductive organ (pistil) in plants is gaining increased attention recently, with most of the previous studies have mainly focused on male gametophyte (pollen) responses (Jagadish 2020). Hybrid rice technology is a viable approach to increase rice production in the world. During hybrid seed production, both male sterile and restorer lines usually flower under hotter summer seasons, causing large reduction in hybrid seed production (Yan et al. 2015). A recent study showed that male sterile lines are more sensitive to high temperature than conventional rice variety when they are exposed to high temperature at flowering for 6 days (Yan et al. 2017), indicating the importance and need for characterizing heat stress effects on female reproductive organ as well as on male reproductive organ (Jagadish 2020). Recent review by Wang et al. (2021), indicated that heat stress differentially impacts pistil development from gametogenesis till embryo formation. In the present study, dusting non-stressed pollen onto heat stressed pistil resulted in significant reductions in spikelet fertility in IR64 at 40 °C, demonstrating the heat sensitivity of the pistil (Fig. 1). But no such reductions in spikelet fertility were noticed in N22, indicating quantifiable genetic differences and varying degree of stress tolerance of pistil to heat stress during development. In contrast to the ambiguous nature of the female reproductive organ, the male reproductive organ (pollen or male gametophyte) has been extensively studied as it is more easily accessible (Wang et al. 2021). Structural and functional abnormalities in pollen leading to failure of fertilization, resulting in spikelet sterility under heat stress has been summarized in rice and other plants (Arshad et al. 2017; Lohani et al. 2020). In the present study, disappearance of megaspore mother cell and degeneration of all four megaspore cells at the early developmental stage in rice exposed to heat stress (Fig. 3), resulted in lesser proportion of viable embryo sac. The abnormalities occurring at the nucleate embryo sac stage, resulted in mature embryo sac without female germ unit, including egg cell and/or polar nuclei (Fig. 4), further affecting fertilization process and seed-set. Similar effect has been reported earlier, wherein heat stress led to degenerated egg and synergids in tomato (Iwahori 1966), improper egg and synergids differentiation in cotton (Snider et al. 2009) and wheat (Saini et al. 1983) affecting fertilization and finally reducing fertility.

Since the polarity of the nuclei is mainly manifested by the regular pattern of positioning of nuclei during embryo sac development, the abnormal positioning of nuclei could distort the polarity of the cell and cause abnormalities, contributing to lower fertility (Zeng et al. 2009). Moreover, these abnormalities were in higher proportion in the sensitive IR64 pistils than the tolerant N22, as N22 had on average 93.9% normal embryo sacs under stress while it was significantly lower (84.4%) in IR64 (Table 1). These results are in accordance with different forms of female reproductive organ abnormalities observed across genotypes/crop species exposed to heat stress (Shahid et al. 2010; Chiluwal et al. 2020). However, one caveat is that the percentage of mature embryo sac with normal structure was more than 80% for both cultivars (Table 1), which was higher than the recorded spikelet fertility (stressed embryo sac pollinated with fresh pollen). This result suggests that pollen fertility together with pollen and pistil interaction, pollen tube growth and subsequent synergid signaling also contribute to spikelet fertility and not just embryo sac fertility (Zeng et al. 2009; Wang et al. 2021).

### Maintenance of Soluble Sugars, Starch, ROS Homeostasis and Antioxidant Activities Promotes Heat Stress Tolerance in N22 Pistils

Plant reproduction relies on adequate supply of carbohydrates, with an insufficient supply leading to restricted development of reproductive organs. A greater decrease in starch in the developing pistil and significantly lower sugars and starch at heading in IR64 compared to N22 which had higher carbohydrate recovery suggests that heat stressed IR64 pistils had insufficient carbohydrate supply for pollination, pollen tube growth and fertilization (Fig. 5). Abundant supply of carbohydrates provided by pistils are important prerequisites for supporting pollen tube growth (Herrero and Hormaza 1996; Snider et al. 2011; Li et al. 2015). Hence, a decline in sugar availability within pistils before pollination could be an important driver inducing high heat sensitivity and decreased spikelet fertility in IR64.

ROS homeostasis in the reproductive organ is crucial for development as it is involved in signaling pathways (Zinta et al. 2016), but higher accumulation of ROS leads to lipid peroxidation, leading to oxidative damage to cells (Dat et al. 2000). The importance of ROS in ovary for normal pollen tube growth has been reported in sweet cherry (Zhang et al. 2018) and sunflower (*Helianthus annuus* L., Sharma and Bhatla 2013), indicating that alternations to ROS levels in the female gametophyte could be an important factor leading to reduced fertility under abiotic stress (Zinta et al. 2016). Immediately after exposure to heat stress, increases in the H_2_O_2_ and MDA and lower levels of antioxidant activity were seen in rice pistils. Oxidative pathway is a highly complicated process and an increase in ROS levels is normally noticed at the initiation of meiosis during sexual reproduction (Hörandl and Hadacek 2013), thus the increased ROS in both the cultivars during female reproductive organ development (6 DAT) may not be fully associated with heat stress response. However, during post-stress recovery at heading, H_2_O_2_ and MDA levels and antioxidant SOD and POD activities were close to control in N22, but the same was not the case with IR64 (Fig. 7), indicating genotypic differences in their recovery to attain ROS homeostasis. A similar increase in H_2_O_2_ and MDA and lower levels of antioxidant activity in pistils at heading/flowering were observed in cotton (Snider et al. 2011), sorghum (Djanaguiraman et al. 2018a) and pearl millet (Djanaguiraman et al. 2018b). In summary, N22 maintained ROS homeostasis and antioxidant activities in female gametophyte better than IR64 especially during the post-stress recovery period, which could play an important role in maintaining spikelet fertility under heat stress.

### Alteration in Female Gametophyte Metabolome Under Heat Stress

**Fig. 10:**
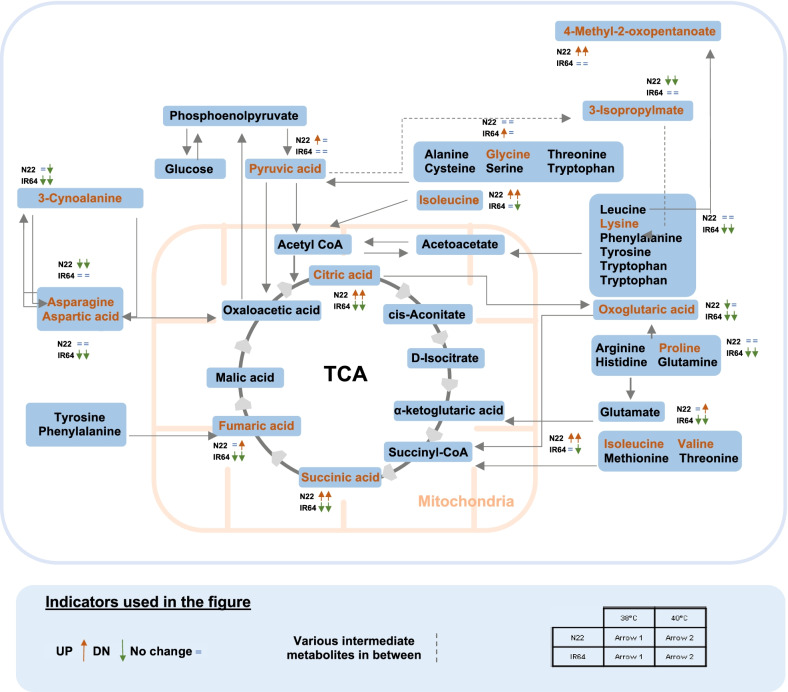
Schematic diagram showing differential abundant metabolites detected in first five highly altered metabolic pathways (including TCA cycle and amino acid pathways) in the two rice genotypes (N22 and IR64) exposed to control and two high temperature treatments (38 °C and 40 °C) for six days during gametogenesis. Up and down arrows indicate regulation based on fold change, while only those metabolites that changed significantly at 5% LSD were included. DN – Down

Metabolomic analysis is one of the important tools used to understand plant mechanisms that operate in response to environmental cues (Razzaq et al. 2019). In this study, 15 metabolites were enriched in five highly altered metabolic pathways including TCA cycle and amino acid metabolisms, which were differentially regulated under heat stress in N22 and IR64 (Fig. 10). Relative change in metabolite levels under heat stress compared to control is more important for thermotolerance than absolute metabolite levels, as metabolic regulations are genotype dependent (Kusano et al. 2007). TCA cycle integrates glucose, amino acid, and lipid metabolisms. In maize, robust metabolic flux through malate metabolic pathway stimulated TCA cycle under heat stress (Qu et al. 2018). In our study, TCA cycle related metabolites i.e., citric acid, fumaric acid and succinic acid recorded a reduction in IR64 under heat stress at gametogenesis while the same were either increased or expressed similar to control in N22 (Figs. 9, 10). Increase in pyruvic acid, the precursor for TCA cycle is known to improve thermotolerance in Arabidopsis (Serrano et al. 2019). Higher accumulation of pyruvic acid noticed in N22 pistil under heat stress aligns with higher total soluble starch accumulation in the ovary. Increase in pyruvic acid level may help in maintaining the TCA cycle and thus, more energy generation. Pyruvic acid also acts as a precursor for the production of 3-Isopropylmate which was found to be less abundant in heat stressed N22 pistils compared to control indicating that most of pyruvic acid is diverted towards TCA cycle during heat stress in the N22 pistil. Whereas, in IR64 no such reduction of 3-Isopropylmate under heat stress was observed after heat exposure. Most of the TCA cycle metabolites including citric acid, oxoglutaric or α-ketoglutaric acid, succinic acid and fumaric acid altered under heat stress were at higher level in N22 pistils particularly at 40 °C indicating the sequential increase of metabolites from pyruvic acid to TCA cycle (Fig. 9). Exogenous application of citric acid is known to have a protective role in maintaining membrane stability, root activity and activation of antioxidant response in tall fescue under heat stress (Hu et al. 2016). In turfgrass, higher accumulation of citric acid helped to minimize heat stress induced damage (Du et al. 2011; Yu et al. 2012). Suppression of mitochondrial citrate synthase (catalyzes the synthesis of citric acid) in potato plants resulted in disintegrated ovaries during flower development (Landschutze et al. 1995). Similarly, application of another TCA cycle intermediate succinic acid with salicylic acid imparted heat tolerance in millets by increasing SOD, catalase, and peroxidase activity (Kolupaev et al. 2011). Thus, accumulation of TCA cycle metabolites during heat stress helps in maintaining ovary structure and ROS homeostasis in N22 pistils exposed to heat stress, while low energy reserves (TCA cycle intermediates) in IR64 pistils, could further reduce the energy available for pollen tube growth (Lohani et al. 2020). This observation is further supported by significantly lower accumulation of starch in IR64 female gametophyte under heat stress (Fig. 5).

In general, amino acid metabolism is highly altered under heat stress (Mayer et al. 1990; Wang et al. 2018). Differential accumulation of amino acids and soluble proteins under heat stress was associated with thermotolerance in different varieties of hard fescue (Wang et al. 2018). In this study, most of the amino acids were significantly decreased in IR64 under heat stress except glycine and asparagine, while the same recorded either a non-significant change or increase under heat stress in N22 (Fig. 10). This indicates that the amino acid synthesis was highly affected in IR64 pistils under stress which can affect protein synthesis. Similarly, in Li et al. (2015), despite heat stress was imposed at a different developmental stage (flowering) and pistil samples were collected just before and after pollination, amino acid metabolism was affected by heat stress. Some of the amino acids including aspartic acid, proline and isoleucine were significantly altered under heat stress in both Li et al. (2015) and this study. Moreover, in Li et al. (2015) sugar metabolism emerged as an important component that determined floral organ tolerance or sensitivity to heat stress at flowering. Proline is a multifunctional amino acid and known to play a regulatory role in thermotolerance (Iqbal et al. 2019; Szabados and Savoure 2010). Proline is also required for pollen development and defects in proline biosynthetic enzymes resulted in sterile pollen grains. (Mattioli et al. 2012). Under heat stress, better maintenance of proline levels in N22 pistil indicates a better regulation of redox homeostasis and stress signaling under heat stress, helping to enhance thermotolerance (Iqbal et al. 2019). Our results provide new insights into the mechanism of pistil adaptation to heat stress at metabolite level and highlight the role of TCA and amino acid metabolism pathways in maintaining thermotolerance as demonstrated in the tolerant N22.

## Conclusions

**Fig. 11:**
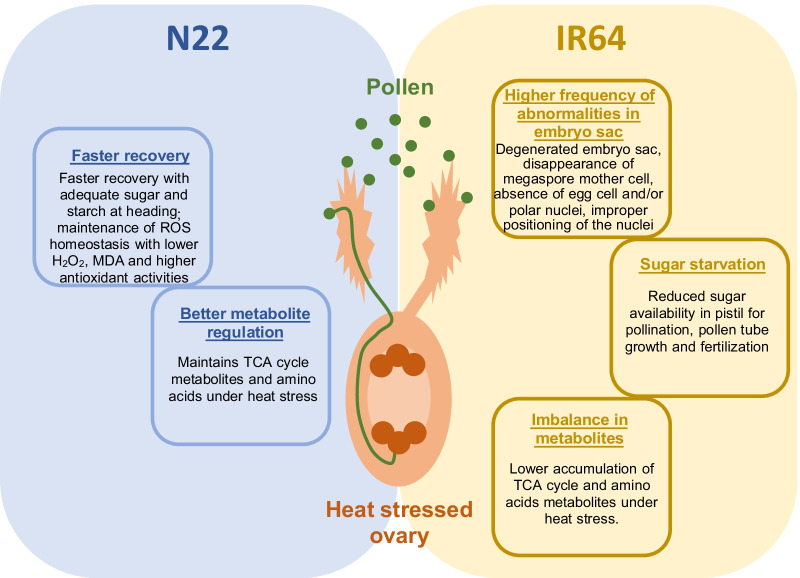
Mechanisms leading to heat sensitivity (reduction in spikelet fertility under heat stress) in IR64 pistil and or processes that allow functional pistil in N22 exposed to heat stress during gametogenesis

Female reproductive organ was sensitive to heat stress, resulting in reduced spikelet fertility in the sensitive genotype IR64. Heat stress induces several abnormalities in embryo sac (Fig. 11). Higher sensitivity of rice genotype IR64 to heat stress was mainly due to higher frequency of abnormalities in embryo sac, sugar starvation, higher ROS accumulation and altered TCA cycle and amino acid metabolites under heat stress (Fig. 11). Heat tolerant N22 exhibited better maintenance of metabolites, a faster recovery through lower H_2_O_2_, MDA, and increased sugar and starch supply. Our findings reveal that heat stress induced damages in female reproductive organ at gametogenesis to be irreversible, leading to spikelet sterility. This study considered the entire pistil but rapid advances made in single cell metabolomics (Shrestha 2020), provides the opportunity for fine scale resolution of mechanisms that induce tolerance to heat stress during gametogenesis. Characterizing mechanisms responsible for enhancing heat tolerance in pistil is crucial for developing crops to better adapt to warming climate. In addition, the findings of this study could have greater significance in hybrid seed production because male sterile lines are highly sensitive to heat stress.

## Methods

### Plant Materials and Growth Conditions

Two rice genotypes, IR64 (sensitive) and N22 (tolerant), with contrasting heat stress responses (Jagadish et al. 2010) were used in the current study. Seeds were sown in seeding trays filled with farm soil after incubating the seeds at 50 °C for 3 days to break seed dormancy. Twenty-day-old seedlings were transplanted into 8-L plastic pots (diameter 22.5 × 29.0 cm height), filled with 9-kg of farm soil mixed with 2-g ammonium sulphate (NH_4_)_2_SO_4_, 1-g potassium chloride (KCl) and 1-g single super phosphate (SSP). An additional 2.5 g of (NH_4_)_2_SO_4_ was top dressed at 25–30 d after transplanting. Plants were grown in a greenhouse with natural sunlight at the Hunan Agricultural University, Changsha (28°11´N, 113°4´E, 29.3 m asl), China. All plants were kept under fully flooded condition to avoid water stress throughout the experiment, including heat stress period. HOBO data loggers (MX2301A, Onset Computer Corp., Bourne, MA, USA) were placed in the greenhouse to record ambient air temperature and relative humidity (RH). The temperature and RH in the greenhouse was 33.9 °C (SD =  ± 2.2) and RH at 73.4% (SD =  ± 5.6) throughout the crop cycle except during the heat stress treatment period.

### Heat Stress Imposition

Main tillers of each genotype were tagged at the panicle gametogenesis stage (− 8 to − 9 cm inter collar distance between the last fully opened leaf and the yet-to-emerge flag leaf) based on an established phenotypic marker (Jagadish et al. 2014) and moved into walk-in growth chambers. Two different heat stress treatments were imposed with one of the heat stress chambers maintained at 38 °C (actual = 37.5 °C [SD ± 0.2]) with a RH of 64.7% ± 2.2 and another at 40 °C (actual = 39.5 °C [SD ± 0.6]) with a RH of 66.2% ± 3.3, from 0830 to 1430 h for six consecutive days as suggested by Jagadish et al. (2014). Simultaneously, two control chambers were maintained at 30 °C (actual = 29.1 °C [SD ± 0.8]) with a RH of 74.5% ± 1.7 during the treatment period. Forty replicate plants per genotype were maintained for each temperature treatment. The night-time temperature (1900–0700 h) was maintained at 24 °C (actual 23.6 ± 1.1[control chambers], 24.8 ± 0.2 [38 °C chamber], 24.3 ± 0.2 [40 °C chamber]) during the treatment period. After six days of heat-stress, plants were moved back to ambient conditions in the greenhouse.

### Emasculation and Manual Pollination

As the plants reached heading stage (about eight days after the end of temperature treatments), unopened spikelets on the tagged main tillers from both control and heat stressed plants were emasculated (anthers were meticulously removed after clipping the tip of the spikelet). Immediately after emasculation, the panicles were covered using glassine bags. Spikelets at the top half of the panicle were emasculated when 50% of the panicle had exerted from the flag leaf node and spikelets at bottom half of the panicle were emasculated on the subsequent day. This was done to make sure that the spikelets were at the same developmental stage during emasculation. Care was taken to remove spikelets that had already flowered at the tip of the panicle (maximum five spikelets) and those that were not fully mature at the lower part of panicle (maximum ten spikelets). This helped to avoid considering naturally sterile spikelets in the lower portions of the panicle or those at the top that would have higher probability to escape the six days of heat stress treatment (Jagadish et al. 2014).

Starting about 10:00 am on the subsequent morning, the emasculated spikelets were pollinated with fresh pollen from a different set of plants maintained under ambient temperature conditions throughout crop growth period. Three batches of 20 pots each for both cultivars were stagger sown to provide a continuous supply of fresh and unstressed pollen grains for manual pollination. Immediately after hand pollination, the target pollinated panicles were re-bagged till seed-set to avoid cross pollination. The pollination process was repeated over two successive days to ensure maximum fresh pollen were deposited on emasculated pistils. In addition to the emasculation and manual pollination in tagged panicles from control and heat stress treatments, another independent set of three plants (about 5 tagged tillers) exposed to heat and control conditions were not emasculated and allowed to pollinate naturally (without manual interference).

### Spikelet Sectioning and Image Analysis

The spikelets with size 4 mm and 6 mm are documented to coincide with gametogenesis stage in N22 and IR64, respectively (Jagadish et al. 2014). To meticulously scrutinize for developmental changes before, during and immediately after gametogenesis, i.e. megasporogenesis and megagametogenesis, spikelets with varying sizes for N22 (3 mm, 4 mm, 5 mm, 6 mm and > 6 mm) and IR64 (5 mm, 6 mm, 7 mm and > 7 mm) from 15 independent main tillers for each treatment were collected at the end of treatment period (6 days after stress). These spikelets were transferred immediately into FAA fixative (50% absolute ethanol, 5% acetic acid, 27% formaldehyde and 18% sterilized water). In addition, spikelets at the heading stage were also sampled for tracking the level of reversibility in morphological changes of the ovary after stress was released.

More than 14 spikelets were sectioned for each spikelet size per genotype and treatment. Briefly, the spikelets were processed by applying a simple and effective eosin B staining procedure and observed by a laser scanning confocal microscope (Zeng et al. 2007). Ovaries from the selected spikelets were carefully dissected and rehydrated in 70%, 50%, 30%, and 10% ethanol and distilled water for 20 min before pretreating in 2% aluminium potassium sulfate for 20 min. They were then stained with 10 mg/L of eosin B solution for 10 to 12 h and subjected to thorough dehydration with distilled water and a series of ethanol solutions (10%, 30%, 50%, 70%, 90%,100%) for 20 min at each concentration. Finally, the ovaries were transferred into absolute ethanol and methyl salicylate (1:1) for 1–2 h, and then kept in pure methyl salicylate for more than 1 h (Shi et al. 2018). The samples were then scanned by a Zeiss laser scanning confocal microscope (LSM 880, Zeiss, Jena, Germany) at excitation wavelength of 543 nm, and emitted light was detected between 550 and 630 nm. The images of developing (gametogenesis) and mature (heading) embryo sac were recorded at 200 × magnification, and the abnormalities were captured.

### Biochemical Analysis

Pistils without anthers, lemma and palea from control and heat stress treatments were obtained at the end of temperature treatments and at heading (before flowering), frozen immediately in liquid nitrogen and stored in − 80 °C freezer for further biochemical analysis.

*Lipid Peroxidation*—was evaluated by measuring thiobarbituric acid reactive substances (TBARS) assay which quantifies the presence of malondialdehyde (MDA). 100 mg frozen pistils were homogenized with liquid nitrogen until fine powder was obtained. 0.5 mL of 0.5% (w/v) thiobarbituric acid in 20% (w/v) trichloroacetic acid and 0.5 mL of buffer (175 mm NaCl in 50 mm Tris–HCl, pH 8) were added and then heated at 95 °C for 30 min. Samples were then immediately cooled in an ice bath and centrifuged at 10,000 rpm for 25 min. Absorbance of 200 μL supernatant was determined at 532 nm and 600 nm (Larkindale and Knight 2002).

*Hydrogen peroxide*—Frozen pistils (10 mg) were homogenized in 3 ml of 10 mM 3-amino-1,2,4-trizole, and then centrifuged for 25 min at 6000 rpm at 4 °C. Then 2 ml of the supernatant was mixed with 1.5 ml 0.1% titanium tetrachloride dissolved in 20% H_2_SO_4_. The reaction mixture was centrifuged and the absorbance of 200 ul supernatant was determined at 410 nm (Brennan and Frenkel 1977).

*Soluble Sugar and Starch Assay—*Total soluble sugar content and starch content in the pistils were quantified using an assay kit (Sino Best Biological Technology Co., Ltd, China, product number YX-W-B602, YX-W-C400). The assay was performed following the manufacturer’s protocol but with 1/5 sample volumes of original protocol. Sugar and starch concentrations were expressed as mg g^−1^ FW.

*Antioxidant Enzyme Activity—*Total SOD (superoxide dismutase) activity in pistils was measured using superoxide dismutase assay kit (Sino Best Biological Technology Co., Ltd, China, product number YX-W-A500). The kit was used to assess superoxide anions (O_2_^−^) which were produced by a xanthine and xanthine oxidase reaction system. The reaction mixture was quantified at 450 nm and one unit of SOD activity is defined as the amount of enzyme required to obtain 50% dismutation of superoxide radical (Sebastiani et al. 2007). The POD (peroxidase) activity was quantified using POD assay kit (Sino Best Biological Technology Co., Ltd, China, product number YX-W-A502), by monitoring the conversion of guaiacol to tetraguaiacol at 470 nm. CAT (catalase) enzyme activity was measured using catalase assay kit (Sino Best Biological Technology Co., Ltd, China, product number YX-W-A501). In the assay, CAT activity was analyzed based on the disappearance of H_2_O_2_ at 240 nm with extinction coefficient (Aebi 1984). One enzyme unit was defined as the amount of catalase enzyme that decomposes 1.0 μmol of H_2_O_2_ min^−1^ from one gram of tissue on a fresh weight basis.

### Metabolite Profiling

The frozen pistils from different spikelet samples (2 genotypes × 3 treatments × 6 biological replicates) were collected on the 6th day of temperature treatment. The spikelets from tagged panicles were immediately frozen in the liquid nitrogen and after removing anthers, lemma and palea, pistils were then carefully dissected on ice, and 8 to 10 pistils at a time were immediately transferred into falcon tubes suspended in liquid N and subsequently stored in − 80 °C for further analysis. This frequent transfer of pistils was followed to ensure that the dissected pistils were immediately frozen, following the method of Li et al. (2015). 50 mg of each sample was used for metabolites extraction and GC–TOF–MS analysis was performed using an Agilent 7890 gas chromatograph coupled with a time-of-flight mass spectrometer following the method of Guo et al. (2015). Raw data preprocessing and annotation, including peak extraction, baseline adjustment, deconvolution, alignment and integration, was done using Chroma TOF (V 4.3x, LECO) software. LECO-Fiehn Rtx5 database was used for metabolite identification by matching the mass spectrum and retention index. Finally, the peaks detected in less than half of quality control (QC) samples or relative standard deviation (RSD) > 30% in QC samples were removed (Dunn et al. 2011). Peak area of representative ion was quantified by MassHunter Quantitation software (Agilent) using the batch specific library (Dhatt et al. 2019). The peak intensity of a selected ion was normalized to that of the internal standard (ribitol).

### Spikelet Fertility

Filled and unfilled spikelets from emasculated panicle and non-emasculated tagged panicles were seperated and manually counted to determine spikelet fertility at physiological maturity. Spikelet fertility was calculated as the ratio of filled spikelets to total number of spikelets.

### Statistical Analysis

Analysis of variance (ANOVA) was carried out for all the physiological data obtained including spikelet fertility, TBARS, total soluble sugar, starch and antioxidant enzymes activity using Genstat (GenStat 16th Edition, Rothamsted Experimental Station, Harpenden, UK). Means were separated using least significant difference (LSD) at *P* ≤ 0.05.

A two-way ANOVA was carried out on the entire normalized metabolite profiling data to identify the significant effect of genotype, treatment and their interactions on metabolite levels using R software. Hierarchical clustering (Euclidean distance) through heatmap was performed in R software (R version 4.0.5) to explore the abundance pattern of metabolites. The differential metabolites were selected on the basis of a combination of a statistically significant threshold of variable influence on projection (VIP) values obtained from the OPLS-DA model (Orthogonal projections to latent structures-discriminant analysis) by using SMICA software (V16.0.2, Sartorius Stedim Data Analytics AB, Umea, Sweden) and p values from a two-tailed Student’s t-test on the normalized peak areas from different groups, where metabolites with VIP > 1.0 and *P* < 0.05 were considered as differential metabolites. Then all differential metabolites were verified manually using Human metabolome database (HMDB), Kyoto Encyclopedia of genes and genomes (KEGG) and PubChem databases. *Oryza sativa* pathway library was used for pathway analysis. The metabolic pathways were identified using Pathway Analysis (MetPA) in a web-based tool MetaboAnalyst platform (https://www.metaboanalyst.ca/).

## Supplementary Information

**Additional file 1: Table S1**. Summary of all observed developing ovaries in this study.

**Additional file 2: Table S2**. Probability values of effects of genotype (G), temperature treatments (T), growth stages (S) and their interaction for various traits.

**Additional file 3: Table S3**. Normalized metabolite profiling data of pistils of two rice genotypes grown under control and heat stress at 6 days after treatment.

**Additional file 4: Table S4**. Probability values of effects of genotype, temperature, and their interaction effects on metabolite levels.

**Additional file 5: Table S5**. Metabolisms with significantly altered metabolites in this study.

## Data Availability

All data generated or analyzed during this study are included in this published article and its Additional information files.

## Abbreviations

CAT: Catalase
DAT: Days after treatment
HD: Heading
HT: Higher temperatures
MDA: Malondialdehyde
MMC: Megaspore mother cell
POD: Peroxidase
ROS: Reactive oxygen species
SOD: Superoxide dismutase
TCA: Tricarboxylic acid cycle
